# Current clinical practice in using adjunctive extracorporeal blood purification in sepsis and septic shock: results from the ESICM “EXPLORATION” survey

**DOI:** 10.1186/s40635-023-00592-6

**Published:** 2024-01-19

**Authors:** Klaus Stahl, Christian Bode, Benjamin Seeliger, Pedro David Wendel-Garcia, Sascha David

**Affiliations:** 1 Department of Gastroenterology, Hepatology, Infectious Diseases and Endocrinology, Hannover Medical School, Carl-Neuberg Straße 1, 30163 Hannover, Germany; 2 Department of Anesthesiology and Intensive Care Medicine, University Hospital Bonn, Bonn, Germany; 3 Department of Respiratory Medicine, Hannover Medical School, Hannover, Germany; 4 Biomedical Research in End-Stage and Obstructive Lung Disease (BREATH), Hannover Medical School (MHH), German Center for Lung Research (DZL), Hannover, Germany; 5 Institute of Intensive Care Medicine, University Hospital Zurich, Zurich, Switzerland; 6 Department of Nephrology, Hannover Medical School, Hannover, Germany

**Keywords:** Sepsis, Septic shock, Blood purification, Hemofiltration, Hemoadsorption, Plasmapheresis, Current status, Research

## Abstract

**Background:**

Despite a lack of clear evidence extracorporeal blood purification (EBP) is increasingly used as an adjunctive treatment in septic shock based on its biological plausibility. However, current state of praxis and believes in both efficacy and level of evidence are very heterogeneous.

**Methods:**

The “EXPLORATION” (Current Clinical Practice in using adjunctive extracorporeal blood purification in septic shock), a web-based survey endorsed by the European Society of Intensive Care Medicine (ESICM), questioned both the current local clinical practices as well as future perspectives of EBP in sepsis and septic shock.

**Results:**

One hundred and two people participated in the survey. The majority of three quarters of participants (74.5%) use adjunctive EBP in their clinical routine with a varying frequency of description. Unselective cytokine adsorption (CA) (37.5%) and therapeutic plasma exchange (TPE) (34.1%) were by far the most commonly used modalities. While the overall theoretical rational was found to be moderate to high by the majority of the participants (74%), the effectively existing clinical evidence was acknowledged to be rather low (66%). Although CA was used most frequently in clinical practice, both the best existing clinical evidence endorsing its current use (45%) as well the highest potential to be explored in future clinical trials (51.5%) was attributed to TPE.

**Conclusions:**

Although the majority of participants use EBP techniques in their clinical practice and acknowledge a subjective good theoretical rationale behind it, the clinical evidence is assessed to be limited. While both CA and TPE are by far the most common used technique, both clinical evidence as well as future potential for further exploration in clinical trials was assessed to be the highest for TPE.

## Background

A dysregulated host response to infection represents the central pathophysiological hallmark of sepsis and septic shock [1]. Current treatment options are still restricted to infection control and supportive measures, such as circulatory support and organ replacement therapies [2]. Although these principles certainly are essential, they do not represent a specific *sepsis therapy*, that would instead have to modulate the cornerstones of the host response consisting of immune alterations, endothelial dysfunction and coagulopathy [1]. Unfortunately, multiple therapeutic approaches, promising in experimental settings and based primarily on modulating *singular* sepsis mediators, have failed to show any survival benefit in clinical trials [3]. While potential reasons for this, including heterogeneity of sepsis phenotypes [4], have often been discussed, doubts remain as to whether modifying a single component in a highly complex pathophysiological network can lead to a relevant improvement in clinical outcome.

Therefore, it is not surprising that the idea of adjunctive extracorporeal blood purification (EBP) to eliminate injurious mediators of sepsis has received increasing interest over the last years [5]. In fact, most likely due to its plausible theoretical rationale, clinical use of EBP techniques, such as hemoadsorption, is described in a multitude of case reports and series [6], despite lack of clear evidence [7, 8]. Moreover, some recent studies have even raised important risk–benefit concerns in employing EBP techniques in critically ill patients [9–11], thus underlining the fundamental need for further research in this field.

Important questions concerning current state of praxis and also the heterogeneity of personal opinions on the evidence for existing and potential future EBP strategies in the treatment of sepsis have not yet been investigated.

“EXPLORATION” (Current Clinical Practice in using adjunctive extracorporeal blood purification in septic shock), a survey endorsed by the European Society of Intensive Care Medicine (ESICM), therefore aimed to survey in a wide range of critical care physicians from different countries both the current clinical practice as well as future perspectives of EBP in sepsis and septic shock.

## Methods

This was an open web-based multi-national survey, endorsed by the ESICM. The survey was posted on the ESICM web-page from 16th of August until 10th of October 2023. No formal invitations were sent out to potential participants. It was not mandatory to be an ESICM member to take part in the survey. The survey aimed only at intensivists; however, a heterogeneous background concerning both subspecialty and training experience was allowed. A total of 102 participants completed the survey. Participants were asked to anonymously answer ten consecutive questions by choosing one out of multiple predefined possible answers (Table 1). In a subset of questions, a specification of user-defined additional response options was possible. The survey was closed after no further participants were recorded for 7 days. All questions were scored and displayed as percentages of the entire participant group, respectively. GraphPad Prism (Version 10.0, GraphPad Software, La Jolla, CA) was used for generation of pie chart graphs.

**Table 1:** Questions and answer possibilities of the “EXPLORATION” survey

Questions	Answer possibilities
Q1: What is your critical care background training?	Medical
	Anesthesia
	Surgical
	Pediatric
	Neurology
Q2: How long have you been working in critical care?	In training
	Completed training and < 10 years’ clinical experience
	Completed training and > 10 years’ clinical experience
Q3: In which ICU setting do you work?	University hospital ICU
	High-performance non-university hospital ICU
	Basic care ICU
Q4: Do you use in your clinical practice extracorporeal blood purification techniques (aside classical renal replacement therapy) as an adjunctive treatment of sepsis or septic shock?	Yes
	No
Q5: How often do you use extracorporeal blood purification techniques a year?	< 5
	5–10
	10–20
	> 20
Q6: What is the most common extracorporeal blood purification technique that you use as an adjunctive treatment of sepsis or septic shock?	High-volume hemofiltration
	Cytokine adsorption (Cytosorb)
	Coupled plasma filtration and adsorption (CPFA)
	oXIRIS
	Therapeutic plasma exchange
	Seraph 100 adsorber
	High-cut-off dialysis
	Polymyxin B hemoperfusion (toramycin)
	Other (please specify):
Q7: How good is the theoretical rationale for using extracorporeal blood purification techniques as an adjunctive treatment of sepsis or septic shock?	1 = very high
	2 = high
	3 = moderate
	4 = low
	5 = very low
	6 = not existing
Q8: How good is the current clinical evidence in general for using extracorporeal blood purification techniques as an adjunctive treatment of sepsis or septic shock?	1 = very high
	2 = high
	3 = moderate
	4 = low
	5 = very low
	6 = not existing
Q9: What is the extracorporeal blood purification technique with the best evidence endorsing its use as an adjunctive treatment of sepsis or septic shock?	High-volume hemofiltration
	Cytokine adsorption (Cytosorb)
	Coupled plasma filtration and adsorption (CPFA)
	oXIRIS
	Therapeutic plasma exchange
	Seraph 100 adsorber
	High-cut-off dialysis
	Polymyxin B hemoperfusion (toramycin)
	Other (please specify):
Q10: What is the extracorporeal blood purification technique most promising for future use as an adjunctive treatment of sepsis or septic shock, that however needs better evidence from RCTs?	High-volume hemofiltration
	Cytokine adsorption (Cytosorb)
	Coupled plasma filtration and adsorption (CPFA)
	oXIRIS
	Therapeutic plasma exchange
	Seraph 100 adsorber
	High-cut-off dialysis
	Polymyxin B hemoperfusion (toramycin)
	Other (please specify):

## Results

### Participants’ critical care background

**Fig. 1:**
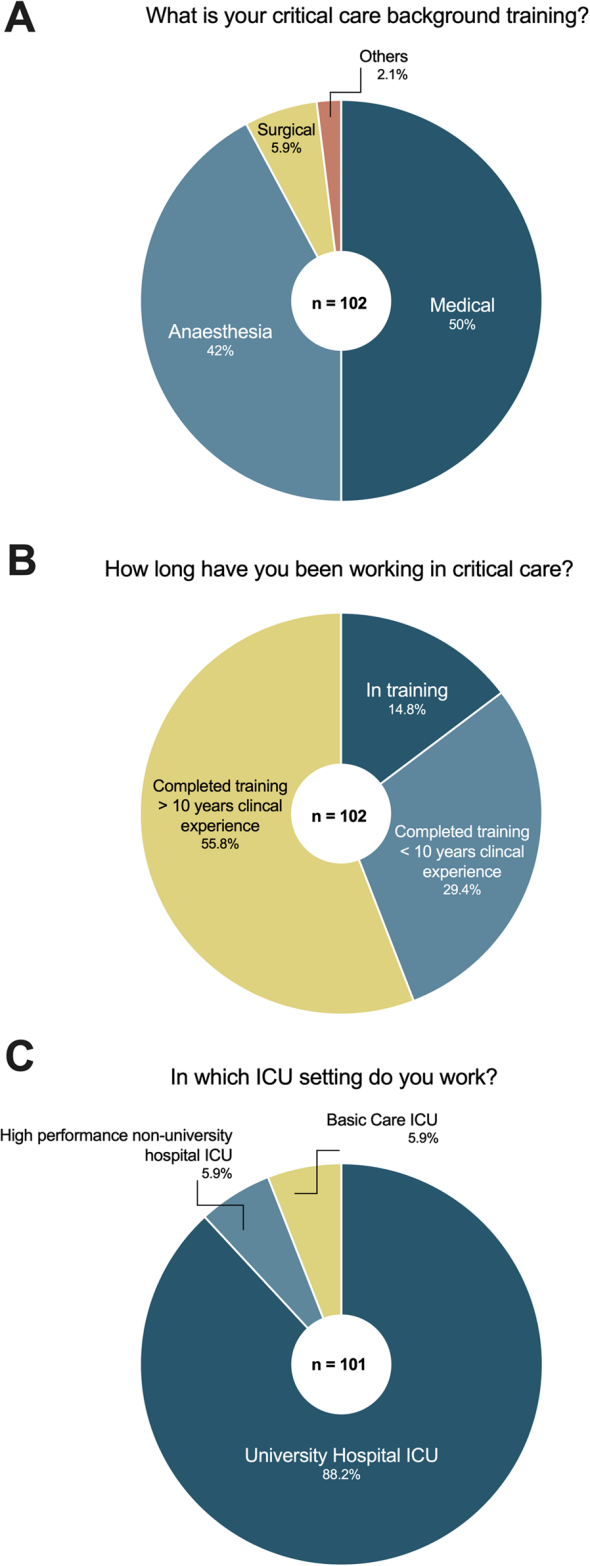
Clinical background of participants

Participants were most commonly working in either a medical (50%) or anesthesiologic (42%) intensive care unit (ICU) with only 5.9% working in a mainly surgical ICU (Fig. 1A). The majority of the respondents (55.8%) had completed training and had a clinical experience of more than 10 years, while only a minority were still in training (14.8%) (Fig. 1B). Eighty-eight percent of participants were employed at a university hospital with only 12% working in a non-university ICU setting (Fig. 1C).

### Current clinical practice in using EBP in sepsis and septic shock

**Fig. 2:**
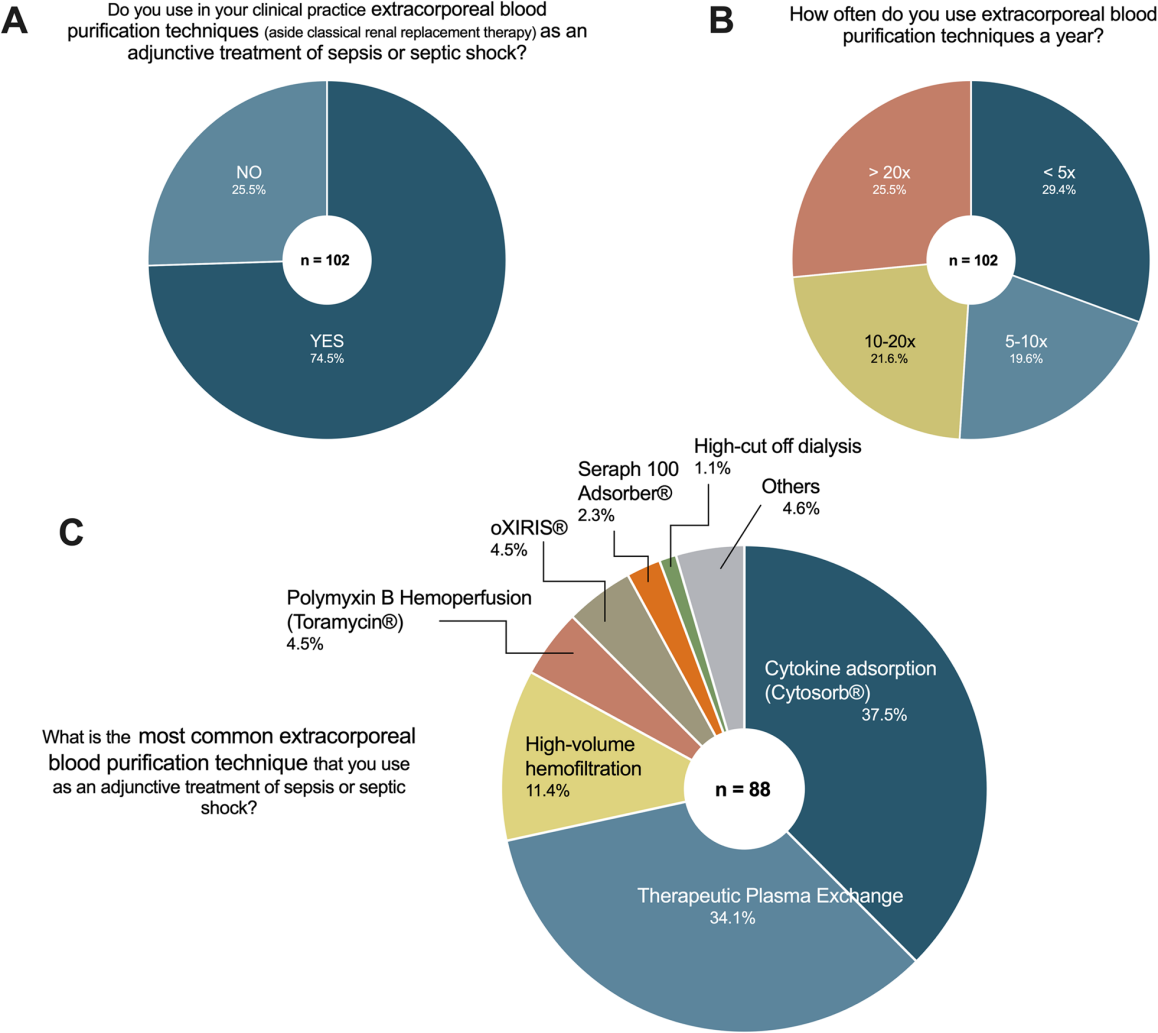
Current clinical practice in applying extracorporeal blood purification techniques in sepsis and septic shock

Most (74.5%) of the participants use EBP in their clinical practice to treat sepsis and septic shock (Fig. 2A). However, the frequency of prescription was heterogeneous: about half prescribed it less than ten times and the other half more than ten times a year (Fig. 2B). The most common used blood purification techniques in clinical practice were cytokine adsorption (CA) (i.e., Cytosorb®) (37.5%) followed by therapeutic plasma exchange (TPE) (34.1%) and high-volume hemofiltration (HVHF) (11.4%) (Fig. 2C). All other EBP modalities were used from less than five percent of the participants.

### Theoretical rationale vs. clinical evidence for different blood purification modalities

**Fig. 3:**
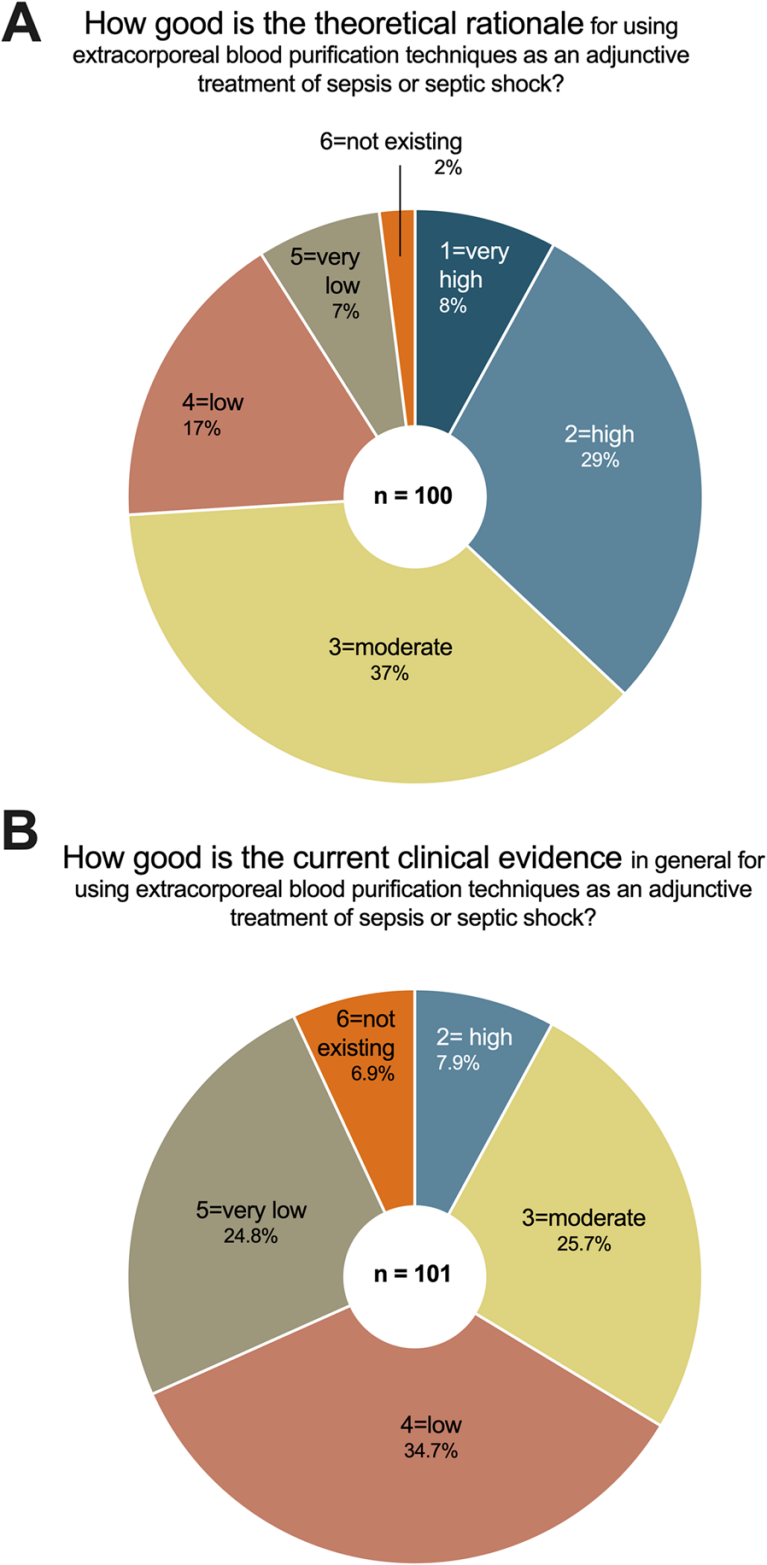
Theoretical rationale and current clinical evidence for applying extracorporeal blood purification techniques in sepsis and septic shock

Seventy-four percent of the participants indicated a moderate to very high theoretical rationale for using EBP techniques in sepsis and septic shock (Fig. 3A). At the same time however, 66% of the respondents assessed the current clinical evidence for using these techniques as non-existing low with only a third indicating a moderate to high clinical evidence (Fig. 3B).

### Preferred blood purification modality in terms of both current clinical evidence and future potential

**Fig. 4:**
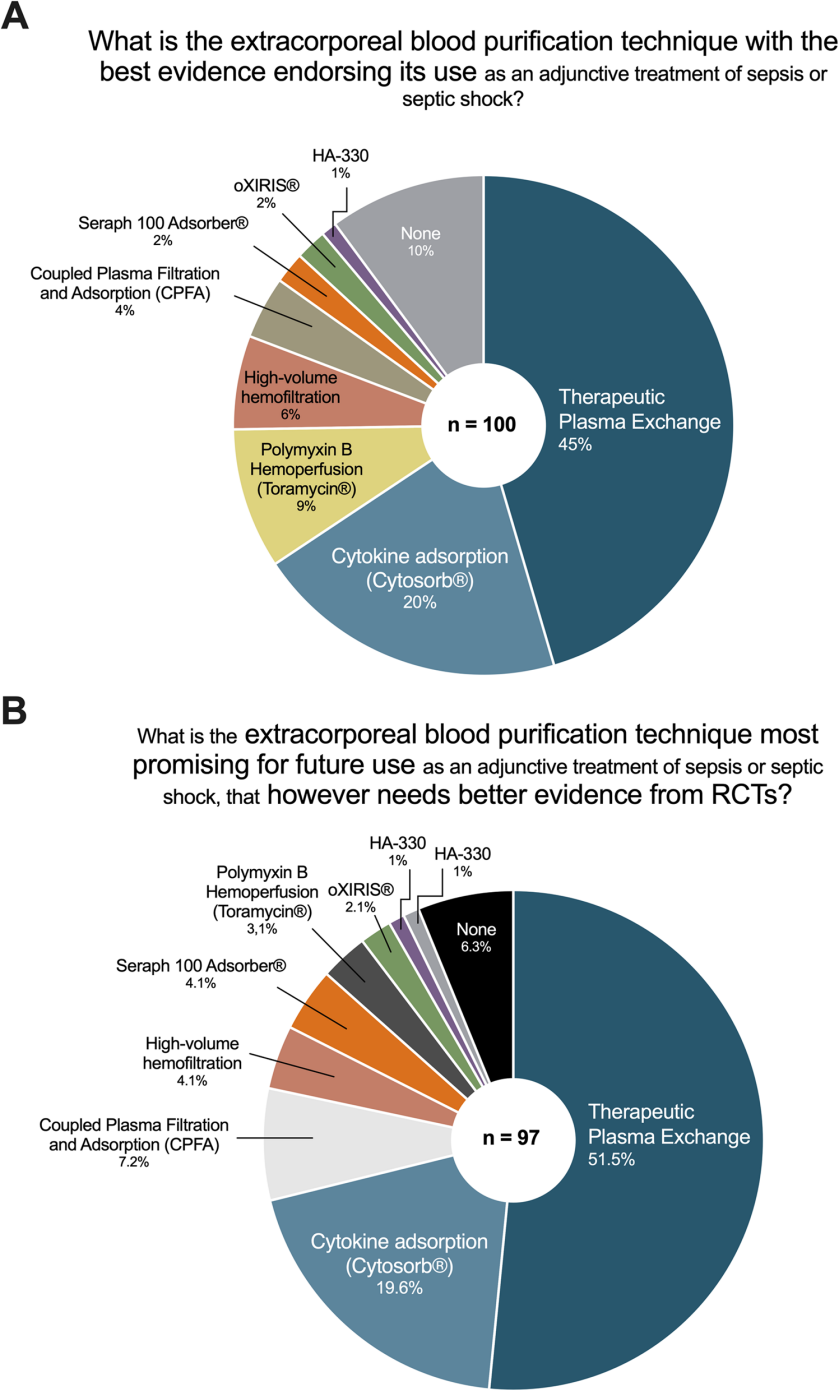
Comparison of different blood purification techniques concerning current evidence and future potential

Blood purification modalities assessed as having the best current evidence for use were TPE (45%), followed by CA (Cytosorb®) (20%), polymyxin B hemoperfusion (Toramycin®) (9%) and HVHF (6%) (Fig. 4A). As most promising for future use and therefore to prioritize in further clinical trials were indicated TPE (51.5%) followed by CA (Cytosorb®) (19.6%) and also coupled plasma filtration and adsorption (CPFA) (7.2%) (Fig. 4B).

## Discussion

This survey, endorsed by the ESICM, evaluated both current clinical practice and the heterogeneity of personal opinions regarding the evidence for existing and potential future EBP therapies in the treatment of sepsis and septic shock. In summary, the majority of approximately three quarters of participants use adjunctive EBP in their clinical routine with a varying frequency of description despite the awareness over the lack of existing evidence.

The results of this survey clearly mirror both the acknowledgment of more recent neutral to negative results from larger randomized clinical trials (RCTs) as well as the promising hypothesis data from other pilot trials. Of note, sufficiently powered RCTs investigating additive use of high-volume hemofiltration [12], polymyxin B hemoperfusion (Toramycin®) [13, 14] and CPFA [9] in septic patients clearly found no survival benefit. Consequentially, these EBP modalities appear to now play only a subordinate role in current clinical reasoning. After promising but underpowered results more than two decades ago [15, 16], TPE has recently been re-investigated, showing improved hemodynamic stabilization in patients with early and severe septic shock [17, 18]. Although recent pooled clinical data suggest potentially improved survival following TPE [19], no positive results from a phase-3 RCT investigating mortality as endpoint are available to the present time. CA using the Cytosorb® device was the most commonly used EBP technique in this survey, despite neutral [7, 8] to negative [10, 11] data even from controlled or propensity score matched trials. Interestingly, both the existing clinical evidence and future potential were assessed to be more than twice as high for TPE than for CA, potentially reflecting the recently appearing uncertainties in evidence.

The survey participants were mostly experienced intensivists working in university-based medical and anesthesiologic ICUs. An important limitation, however, is the almost absence of surgical intensivists responding to the survey, potentially restricting generalizability of the survey results. Nevertheless, in many centers anesthesiologic intensivists care for post-surgical critically ill patients. The almost absence of participants working in non-university ICU settings as well as the majority of respondents supporting in general use of EBP represent further potential selection bias of this study. Since this open survey was posted online at the ESICM website without any further formal invitations sent out, it is not possible to adequately determine a response rate.

## Conclusions

The majority of participants use blood purifications techniques in their clinical practice most likely driven by a plausible theoretical rationale despite the awareness of lack of clinical evidence. While both CA and TPE are by far the most commonly used techniques, both clinical evidence and the potential for further research in clinical trials were surveyed to be highest for TPE.

## Data Availability

The datasets used and analyzed are during the current study are available from the corresponding author on reasonable request.
